# Incorporating Physical Activity in a New Two-Oscillator Model of Circadian Activity in Nocturnal and Diurnal Mammals

**DOI:** 10.1177/07487304241303554

**Published:** 2024-12-26

**Authors:** Anouk W. van Beurden, Johanna H. Meijer, Jos H. T. Rohling

**Affiliations:** Department of Cell and Chemical Biology, Leiden University Medical Center, Leiden, The Netherlands

**Keywords:** circadian rhythm, suprachiasmatic nucleus, Poincaré model, physical activity, nocturnality, diurnality

## Abstract

In both diurnal and nocturnal species, the neurons in the suprachiasmatic nucleus (SCN) generate a daily pattern in which the impulse frequency peaks at midday and is lowest during the night. This pattern, common to both day-active and night-active species, has led to the long-standing notion that their functional difference relies merely on a sign reversal in SCN output. However, recent evidence shows that the response of the SCN to the animal’s physical activity is opposite in nocturnal and diurnal animals. This finding suggests the presence of additional differences in the circadian system between nocturnal and diurnal species. We therefore attempted to identify these differences in neuronal network organization using the A-B two-oscillator model, which is comprised of Poincaré like oscillators. Based on this model, we infer that in diurnal animals the feedback from physical activity acts on neuronal subpopulations in the SCN that do not receive light input; in contrast, in nocturnal animals, physical activity acts on light-receptive neurons in the SCN in order to produce high-amplitude circadian rhythms.

Mammals have evolved an internal “clock” that enables them to adapt to daily changes in their environment (Hastings et al., 2018; Takahashi, 2017). The endogenous clock, which is located in the suprachiasmatic nucleus (SCN) of the hypothalamus, is entrained to the external light-dark cycle by light input. The SCN is essential for regulating the timing of the animal’s physical activity (PA) over a 24-h time period. Some species are active primarily during the daytime (i.e., diurnal), while others are active primarily during the night (i.e., nocturnal) (Challet, 2007). The SCN produces a rhythm of approximately 24 h, characterized by high neuronal activity during the day and relatively low neuronal activity during the night, in both nocturnal and diurnal species (Meijer et al., 1997; Bano-Otalora et al., 2021). Thus, SCN activity is inversely related to physical activity in nocturnal animals.

Recordings of the SCN in freely moving animals have revealed that physical activity in turn provides feedback to the clock (Hughes and Piggins, 2012; Jha et al., 2021). In nocturnal rodents, physical activity acutely suppresses the SCN’s impulse frequency (Schaap and Meijer, 2001; van Oosterhout et al., 2012). Because nocturnal animals are active during the trough in SCN impulse frequency, physical activity at night lowers the trough of the rhythm. In contrast, the SCN’s impulse frequency in diurnal rodents is increased by physical activity (Caputo et al., 2024); consequently, daytime activity in diurnal animals raises the peak in SCN impulse frequency. Thus, the amplitude of the SCN’s rhythm can be enhanced by appropriately timed physical activity, by either a lowering of the trough during the night or a higher level of electrical activity during the day.

Many processes in the SCN have a similar phase between diurnal and nocturnal animals, including their rhythmic patterns of gene expression. Consequently, the major differences between diurnality and nocturnality have previously been attributed to a phase reversal downstream of the SCN (Smale et al., 2003; Mrosovsky, 2003). However, the differential effects in feedback from physical activity to the SCN suggest that additional, unidentified differences in the SCN neuronal network could exist between nocturnal and diurnal animals. Both physical activity and light input to the SCN are known to affect only a subpopulation of neurons (Abrahamson and Moore, 2001). Whether these subpopulations of recipient neurons overlap—either partly or fully—or are distinct subpopulations is unknown.

Here, we investigated the network properties of the SCN based on the effect of feedback from physical activity to the light-entrained SCN. We used a two-oscillator model, which is comprised of Poincaré like oscillators to simulate the SCN. Specifically, we used distinct network configurations in order to obtain new insights into the network differences between the diurnal and nocturnal SCN. Given the beneficial influence of physical activity on the SCN’s amplitude in both nocturnal and diurnal mammals, we infer from our model that feedback from physical activity acts on light-receptive neuronal subpopulations in the nocturnal SCN, but acts on non-light-receptive neuronal subpopulations in the diurnal SCN.

## Materials and Methods

### Simulations of the SCN

All numerical simulations were performed using a Poincaré model. The Poincaré model is often used to describe the SCN’s oscillatory network (Bordyugov et al., 2011; Gu et al., 2016; Schmal et al., 2020; Zhou et al., 2022), because it provides a simple mathematical framework containing amplitude and phase information (Abraham et al., 2010). As we will perform amplitude manipulations, the Poincaré model is especially suited in this study, compared to for instance the Kuramoto model which only contains phase information. We first used a single-oscillator Poincaré model to determine whether the model can reproduce the empirical data showing that in nocturnal animals the trough of the SCN rhythm is lowered by PA (van Oosterhout et al., 2012), while in diurnal animals the peak of the SCN rhythm is elevated by PA (Caputo et al., 2024). In all simulations, we considered the condition in which the animal is exposed to a 12 h:12 h light-dark cycle (see supplemental figures for other photoperiods). We defined an animal as nocturnal when it receives light and PA input to the SCN at opposing times (i.e., light input during the day and PA input during the night) and diurnal when it receives concurrent light and PA input to the SCN (i.e., both light and PA input during the day, with no input during the night). Next, we applied a two-oscillator model, as both PA and light are known to affect only a subpopulation of SCN neurons (Abrahamson and Moore, 2001). We used 2 different two-oscillator network configurations to determine whether or not these subpopulations overlap.

### Description of Poincaré Model

In the Poincaré model, each neuron is represented by 2 variables, *x* and *y*, which contain amplitude and phase information.

(1) $$\begin{array}{c} \frac{dx_{i}}{dt}=\lambda x_{i}\left(a-r_{i}\right)-\frac{2\pi }{\tau }y_{i}+gF+L_{i}+PA_{i},\\ \frac{dy_{i}}{dt}=\lambda y_{i}\left(a-r_{i}\right)+\frac{2\pi }{\tau }x_{i}, \end{array}$$

where the subscript *i* represents the *i*th oscillator (*i* = 1, . . ., *N*), and the parameters λ, *a*, and τ represent the relaxation rate, intrinsic amplitude, and intrinsic period of the neuronal oscillator, respectively. The individual oscillators are coupled via the term *gF*, where *g* represents the coupling strength and *F* is the mean-field value of the variable *x*. This type of coupling represents the global diffusion of neurotransmitter (Locke et al., 2008). Finally, *r_i_* represents the amplitude of a single oscillator. *r_i_* and *F* are defined as follows:

(2) $$r_{i}=\sqrt{x_{i}^{2}+y_{i}^{2}},$$

(3) $$F=\frac{1}{N}\overset{N}{\underset{i=1}{\sum }}x_{i},$$

*L_i_* represents the input of light and *PA*_i_ represents the input from PA, as follows:

(4) $$L_{i}=\{\begin{array}{ccc} K_{L,} & if\;mod\left(t,T\right)<T/2 & \left(i\;\in LS\right)\\ 0, & \; & \left(otherwise\right), \end{array}$$

(5) $$PA_{i}=\{\begin{array}{ccc} dK_{PA}, & \mathrm{if}\;\mathrm{mod}\left(t,T\right)<T/2 & \left(i\;\in PAS\right)\\ nK_{PA}, & \mathrm{if}\;\mathrm{mod}\left(t,T\right)\geq T/2 & \left(i\;\in PAS\right)\\ 0, & \; & \left(\mathrm{otherwise}\right) \end{array},$$

where *T* represents the period of the external light-dark cycle, *K_L_* represents the light intensity of *L_i_*, and *LS* represents the group of light-sensitive neurons (equation (4)). *K_PA_* represents the strength of behavioral feedback of *PA_i_*, and *PAS* represents the group of PA-sensitive neurons. When representing a diurnal animal, *d* = 1 and *n* = 0; when representing a nocturnal animal, *n* = 1 and *d* = 0 (equation (5)).

### Network Configurations

**Figure 1.**
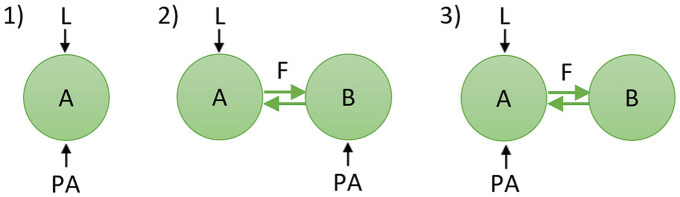
Schematic diagram depicting the network configurations used in this study. The green circles represent subpopulations of neurons (i.e., oscillators) in the SCN. The black arrows represent light (L) and physical activity (PA) input to the SCN, and the green arrows in the two-oscillator configurations represent coupling (F) between the 2 subpopulations.

We examined 1 single-oscillator configuration and 2 two-oscillator configurations (Figure 1). The first configuration (1) consisted of a single oscillator (“A”) that is both light-receptive and PA-receptive (i.e., multiple identical oscillators). The other 2 configurations consisted of 2 coupled oscillators (oscillators “A” and “B”) that represent functional neuronal subpopulations in the SCN and are coupled to each other using a mean-field term. In the second configuration (2), one oscillator (A) is light-receptive, and the other oscillator (B) is PA-receptive. Finally, in the third configuration (3), one oscillator (A) is both light-receptive and PA-receptive, and the other oscillator (B) does not receive any external input.

### Definition of Amplitude and Phase

The amplitude-increasing and phase-shifting effects of PA input to the light-entrained SCN were investigated for all network configurations. We used the time trace(s) of variable *x* to determine amplitude and phase. The amplitude was defined as the absolute difference in height between the peak and the trough of the rhythm. To determine the phase of the rhythm relative to the light-dark cycle, we expressed the peak of the rhythm in zeitgeber time (ZT), where ZT12 corresponds with the time of lights-off. We consider the rhythm to be properly entrained to the light-dark cycle when the peak of the oscillatory rhythm occurs between ZT3 and ZT9.

### Simulation Details

We selected our parameter values based on the parameter optimization performed by Tokuda et al. (2020). The following ranges of parameter values were used: λ ∈[0.35,0.45] , a∈[1.2,2.1] , τ = ∈[23.5,24.5] , *g* ∈[0,0.1] , *K*_*L*_ ∈[0.5,1.0] , *K*_*PA*_ ∈[−1.5,1.5] . Note that *g* *=* 0, for the single-oscillator configuration, because there is no self-feedback, and *g* = 0.1 for the two-oscillator configurations. λ was selected in the upper part of the range given by Tokuda et al. (2020) because Tokuda and colleagues only considered light input to the SCN and here we have the additional input of PA to the SCN. The amplitude-enhancing effects of PA are acute and only last as long as the animal is active (Caputo et al., 2024; van Oosterhout et al., 2012), which can be reflected by a relatively faster amplitude relaxation (i.e., more robust oscillator). Negative values for *K_PA_* indicate inhibitory input and positive values indicate excitatory input. In the model, the initial conditions of the variables *x_i_* and *y_i_* were randomly selected from a uniform distribution ranging from 0 to 1. We used MATLAB’s ode45 with error control for the numerical simulations. The integration was stopped and re-started at the times of discontinuities in the inputs *L* and *PA*, that is every 12 h, using ordinary differential equation (ODE) Event Locations. To avoid the potential effects of transients, the initial 1.2 × 10^4^ h was neglected. The simulations were performed in MATLAB version 2020a (MathWorks, Natick, MA).

## Results

### The Single-Oscillator Model

**Figure 2.**
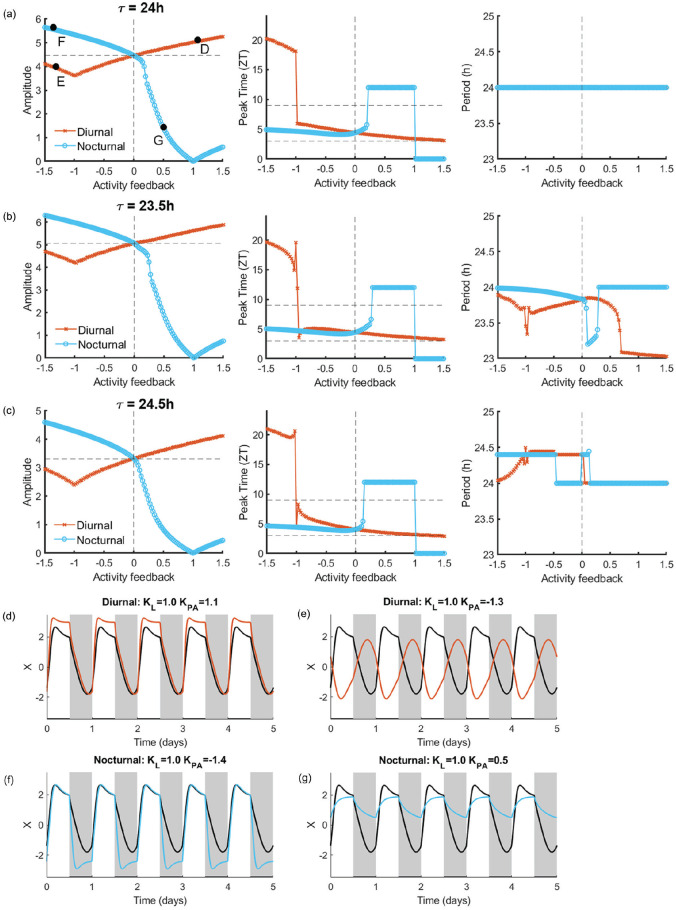
Amplitude and phase depend on the strength of physical activity feedback. (a) Left: the amplitude of the circadian rhythm in the SCN with τ = 24 h, *a* = 1.8, and λ = 0.4, of diurnal (shown in orange) and nocturnal (shown in blue) animals under various strengths of physical activity feedback. The negative and positive values of physical activity indicate inhibitory and excitatory input, respectively. The horizontal dashed line indicates the amplitude of the rhythm without physical activity feedback (indicated by the vertical dashed line). Middle: the corresponding phase of the rhythm is indicated by the peak time given in zeitgeber time. The horizontal dashed lines demark the range of the zeitgeber time for which the rhythm is properly entrained to the light-dark cycle. Right: the corresponding period of the rhythm. (b) Same as (a) for τ = 23.5 h, *a* = 2.1, and λ = 0.35. (c) Same as (a) for τ = 24.5 h, *a* = 1.2, and λ = 0.45. (d-g) Example time traces for a single oscillator under the conditions indicated by the solid black circles in panel a. In these graphs, gray and white correspond to darkness and light, respectively, and the black bars at the bottom indicate the timing of physical activity. In each graph, the black trace indicates the baseline rhythm measured using only light input (*K_L_* = 1.0).

To validate the use of a Poincaré model to simulate PA as input to the SCN in addition to light, we compared the results of the simulations with our previously published empirical data (van Oosterhout et al., 2012; Caputo et al., 2024). The amplitude of the rhythm from the single-oscillator model together with the corresponding phase and period are shown in Figure 2a-2c for various strengths of PA feedback (see Figure S1 for changes in photoperiod); in Figure 2a τ = 24 h, while in Figure 2b τ < 24 h and in Figure 2c τ > 24 h. We consider the rhythm to be properly entrained to the light-dark cycle when the period of the rhythm is 24 h and the peak of the rhythm falls in between ZT3 and ZT9.

In the case that PA feedback is excitatory in diurnal animals, the height of the peak of the rhythm increases in proportion to the strength of the PA feedback, and the rhythm shows the proper phase of entrainment with respect to the light-dark cycle (example 2d). For τ = 24 h and τ > 24 h, there is entrainment to the external light-dark cycle, while for τ < 24 h there is no entrainment to the external light-dark cycle, due to the rhythm not being 24 h periodic. In contrast, when PA feedback is inhibitory in diurnal animals, 2 scenarios are possible. In the first scenario, in which |*K_PA_*| < *K_L_*, the net effect is excitatory; the rhythm shows the proper phase of entrainment, albeit with a lowered amplitude. There is only proper entrainment to the external light-dark cycle for τ = 24 h. In the second scenario, in which|*K_PA_*| > *K_L_*, the net effect is inhibitory; the amplitude of the rhythm is slightly decreased, and the rhythm is phase-shifted into antiphase (example 2e).

For the conditions in which the nocturnal SCN is simulated, the situation is reversed. When PA feedback is inhibitory in nocturnal animals, the trough of the rhythm lowers in proportion to the strength of PA feedback, and the rhythm shows the proper phase of entrainment with respect to the light-dark cycle (example 2f). There is entrainment to the external light-dark cycle for τ = 24 h, for τ < 24 h with strong activity feedback, and for τ > 24 h with weak activity feedback. When PA feedback is excitatory in nocturnal animals, there is a continuous excitatory input, causing a major reduction in the rhythm amplitude (example 2g).

Thus, the amplitude-enhancing effects of PA in our model depend on the strength of the PA input and only occur when the PA feedback is excitatory in the diurnal SCN and inhibitory in the nocturnal SCN, which is consistent with the empirical data (van Oosterhout et al., 2012; Caputo et al., 2024). These results therefore support our use of a Poincaré model to simulate PA input to the light-entrained SCN.

### Two-Oscillator Model

**Figure 3.**
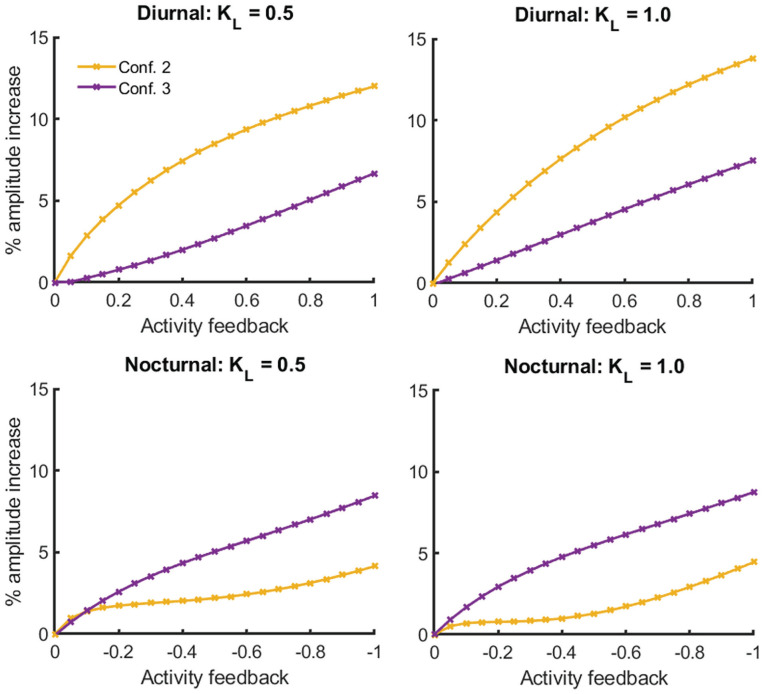
The activity feedback‒induced increase in amplitude depends on the network configuration. In each panel, the increase in amplitude (%) is plotted for both two-oscillator network configurations (see Figure 1) in diurnal (top row) and nocturnal (bottom row) animals for 2 levels of light intensity (“*K_L_*”).

To investigate whether network differences exist in the SCN between diurnal and nocturnal animals based on PA input, we used 2 different two-oscillator configurations for our model (see Figure 1). In simulations representing diurnal and nocturnal animals, we used only excitatory or inhibitory PA feedback, respectively, in the two-oscillator model, as these conditions were found to be optimal in the single-oscillator model and are consistent with our empirical data (van Oosterhout et al., 2012; Caputo et al., 2024). The PA-induced increase in rhythm amplitude is shown in Figure 3 for the 2 network configurations for both diurnal and nocturnal animals. The following parameter values were used: τ = 24, *a* = 1.8, and λ = 0.4 (see Figure S2 for changes in photoperiod). To evaluate the amplitude increase, we used the condition with light input, but without PA input as a control. We observed an increase in amplitude for all conditions in which PA input was included.

In diurnal animals, the largest increase in amplitude occurs when one oscillator is light-receptive and the other oscillator is PA-receptive (the second configuration in Figure 1), whereas the smallest increase in amplitude occurs when one oscillator is both light-sensitive and PA-receptive and the other oscillator does not receive any external inputs (the third configuration in Figure 1). As light intensity increases, the increase in amplitude becomes larger for the second configuration.

In nocturnal animals, the largest increase in amplitude can be achieved with the third configuration, in which the first oscillator is both light-receptive and PA-receptive. The smallest amplitude increase is achieved when one oscillator is light-receptive and the other oscillator is PA-receptive. By increasing light intensity, the increase in amplitude is larger for the third configuration. Thus, increasing light intensity has the exact opposite effect in nocturnal animals compared to diurnal animals.

## Discussion

Using a modified Poincaré model, we simulated physical activity feedback as input to the light-entrained SCN in diurnal and nocturnal species. With the two-oscillator model, we captured the physical activity feedback‒induced increase in rhythm amplitude, consistent with our experimental observations (van Oosterhout et al., 2012; Caputo et al., 2024). The optimal network configuration appears to differ between the diurnal SCN and the nocturnal SCN. In the diurnal SCN, feedback from physical activity acts on non-light-receptive neuronal subpopulations to achieve the largest amplitude increase; in contrast, in the nocturnal SCN, physical activity acts on light-receptive neuronal subpopulations. These results are robust to changes in photoperiod.

An unexpected outcome of our simulations was that for diurnal animals their intrinsic period should be 24 h, or longer than 24 h, in order to entrain to the external light-dark cycle. In some conditions, amplitude changes can induce period changes in nonlinear oscillators, also referred to as twist (Del Olmo et al., 2023).

In mice, the physical activity induced increase in rhythm amplitude depends on the type of behavior, with more intense types of behavior resulting in a larger amplitude increase (van Oosterhout et al., 2012). This fits nicely with our model in which stronger physical activity feedback also results in a larger amplitude increase. Yet, we have decided to make a qualitative comparison with the empirical data rather than a quantitative comparison. We did this because the amplitude as well as the amplitude manipulation of the model depends on the chosen parameter values. According to limit cycle theory, the amplitude expansion depends on the amplitude relaxation rate (Abraham et al., 2010; Pavlidis, 1969), and it is not possible to determine the amplitude relaxation rate experimentally.

The amplitude of the SCN’s impulse frequency rhythm is increased in both diurnal and nocturnal animals only when exercise is properly timed, meaning that a diurnal animal is active only during the day, while a nocturnal animal is active only during the night. In diurnal animals, both light and physical activity have excitatory effects. Based on the empirical data, increasing the rhythm amplitude requires both light and physical activity to be present simultaneously. In nocturnal animals, light input is excitatory, whereas physical activity input is inhibitory. Therefore, both light input and physical activity input must occur at different times in the circadian cycle (i.e., out of phase with each other), or they would cancel each other out. Note that for physical activity, we used 12-h blocks of activity followed by 12-h blocks of inactivity. If an animal engages in a small amount of activity during “incorrect timepoints” in real-life conditions, the animal’s SCN would not immediately become unstable; indeed, the SCN would be affected only if large amounts of activity occur during the “incorrect” time.

For our two-oscillator model, we performed simulations using 2 distinct network configurations, in which always only one subpopulation was PA-receptive. Experimental studies performing multiunit activity recordings in rats (Schaap and Meijer, 2001), mice (van Oosterhout et al., 2012), and arvicanthis (Caputo et al., 2024) have shown that not all subpopulations in the SCN are responsive to physical activity. In rats, the proportion of neurons that are PA-receptive is smaller than the proportion of neurons that are light-receptive (Schaap and Meijer, 2001). Thus, visual input may be stronger than behavioral input in nocturnal species, but not necessarily in diurnal animals.

Light input reaches the SCN via the retinohypothalamic tract (Morin and Allen, 2006), whereas physical activity input reaches the SCN via one of the major nonphotic input pathways, consisting of afferents from the raphe nucleus and the intergeniculate leaflet (Morin, 2013). These nonphotic input pathways are better studied in nocturnal rodents than in diurnal rodents; in nocturnal rodents, overlap exists between the areas of the SCN that are innervated by the photic pathways and areas innervated by the nonphotic input pathways, as both pathways project primarily to the ventrolateral part of the SCN (Harrington, 1997; Challet and Pévet, 2003). Therefore, it is reasonable to speculate that physical activity can act on light-receptive neuronal subpopulations in the nocturnal SCN, as predicted by our model. For diurnal rodents, it remains to be elucidated whether the prediction based on the model—namely, that light and physical activity innervate distinct neuronal subpopulations within the SCN—is correct.

The “A” and “B” oscillators were first introduced by Pittendrigh et al. (1958), as 2 distinct oscillating systems. The “A” oscillator was light-sensitive and self-sustained, while the “B” oscillator was driven by “A” and temperature-sensitive. Later, the “A” and “B” oscillators have been used to represent dusk and dawn (Daan and Berde, 1978), and the core and shell SCN (for review, see Evans and Schwartz, 2024). Our “A” and “B” oscillator model loosely reflects the model of Pittendrigh, insofar that only the “A” oscillator is light-sensitive.

Arguably, both the “A” and “B” subpopulations can be present in the SCN; alternatively, the SCN may contain only one of them. In all configurations, the “A” oscillator is depicted as light-receptive and is therefore responsible for entraining the SCN to the external light-dark cycle. This property makes it highly likely that the “A” oscillator is actually located within the SCN. The “B” oscillator in our model, on the other hand, is either PA-receptive or does not receive any external input signals. When PA-receptive, the “B” oscillator tunes the amplitude of the ensemble rhythm. This tuning of the rhythm amplitude could occur either within the SCN itself or it could be established via communication with surrounding brain areas; therefore, the “B” oscillator does not necessarily need to be located within the SCN.

In this modeling study, we assumed that the light input to the SCN is excitatory. In nocturnal rats, hamsters (Meijer et al., 1986), and mice (Schoonderwoerd et al., 2022), the vast majority of responses to light in the SCN are indeed excitatory. In contrast, studies in diurnal squirrels (Meijer et al., 1986), degus (Jiao et al., 1999), and *Rhabdomys* (Schoonderwoerd et al., 2022) suggest that responses to light in the diurnal SCN are equally excitatory and inhibitory. Yet, in both nocturnal and diurnal animals, the phase response curve for light is similar and mediated by the excitatory part of the light responses (Novak et al., 2008). As we used light input only to model entrainment to the external light-dark cycle, we took notice of only the excitatory light input in both nocturnal and diurnal animals.

**Figure 4.**
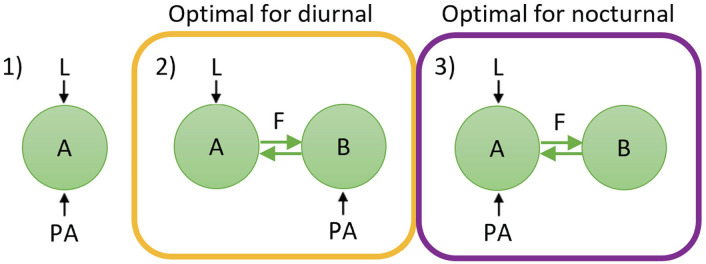
Overview of the network configurations. Configuration 2 is optimal for diurnal animals, while configuration 3 is optimal for nocturnal animals.

Physical activity can increase the rhythm amplitude of impulse frequency in the SCN; however, both the timing of physical activity and the response of the SCN to physical activity differ between diurnal and nocturnal animals. Therefore, a certain degree of asymmetry in the SCN’s wiring must exist between the diurnal SCN and the nocturnal SCN in order to integrate the input signals from light and physical activity beneficially (Figure 4). For diurnal animals, the input signals are optimally integrated when light and physical activity act on different subpopulations in the SCN (configuration 2), whereas for nocturnal animals, the input signals are optimally integrated when light and physical activity act on the same subpopulation in the SCN (configuration 3).

## Supplemental Material

sj-tif-1-jbr-10.1177_07487304241303554 – Supplemental material for Incorporating Physical Activity in a New Two-Oscillator Model of Circadian Activity in Nocturnal and Diurnal Mammals Supplemental material, sj-tif-1-jbr-10.1177_07487304241303554 for Incorporating Physical Activity in a New Two-Oscillator Model of Circadian Activity in Nocturnal and Diurnal Mammals by Anouk W. van Beurden, Johanna H. Meijer and Jos H. T. Rohling in Journal of Biological Rhythms

sj-tif-2-jbr-10.1177_07487304241303554 – Supplemental material for Incorporating Physical Activity in a New Two-Oscillator Model of Circadian Activity in Nocturnal and Diurnal Mammals Supplemental material, sj-tif-2-jbr-10.1177_07487304241303554 for Incorporating Physical Activity in a New Two-Oscillator Model of Circadian Activity in Nocturnal and Diurnal Mammals by Anouk W. van Beurden, Johanna H. Meijer and Jos H. T. Rohling in Journal of Biological Rhythms
