# Supplementary figures and images for: Incorporating Physical Activity in a New Two-Oscillator Model of Circadian Activity in Nocturnal and Diurnal Mammals
Source: J Biol Rhythms. 2024 Dec 26;40(1):27–35. doi: 10.1177/07487304241303554 (PMC11834329; doi:10.1177/07487304241303554)

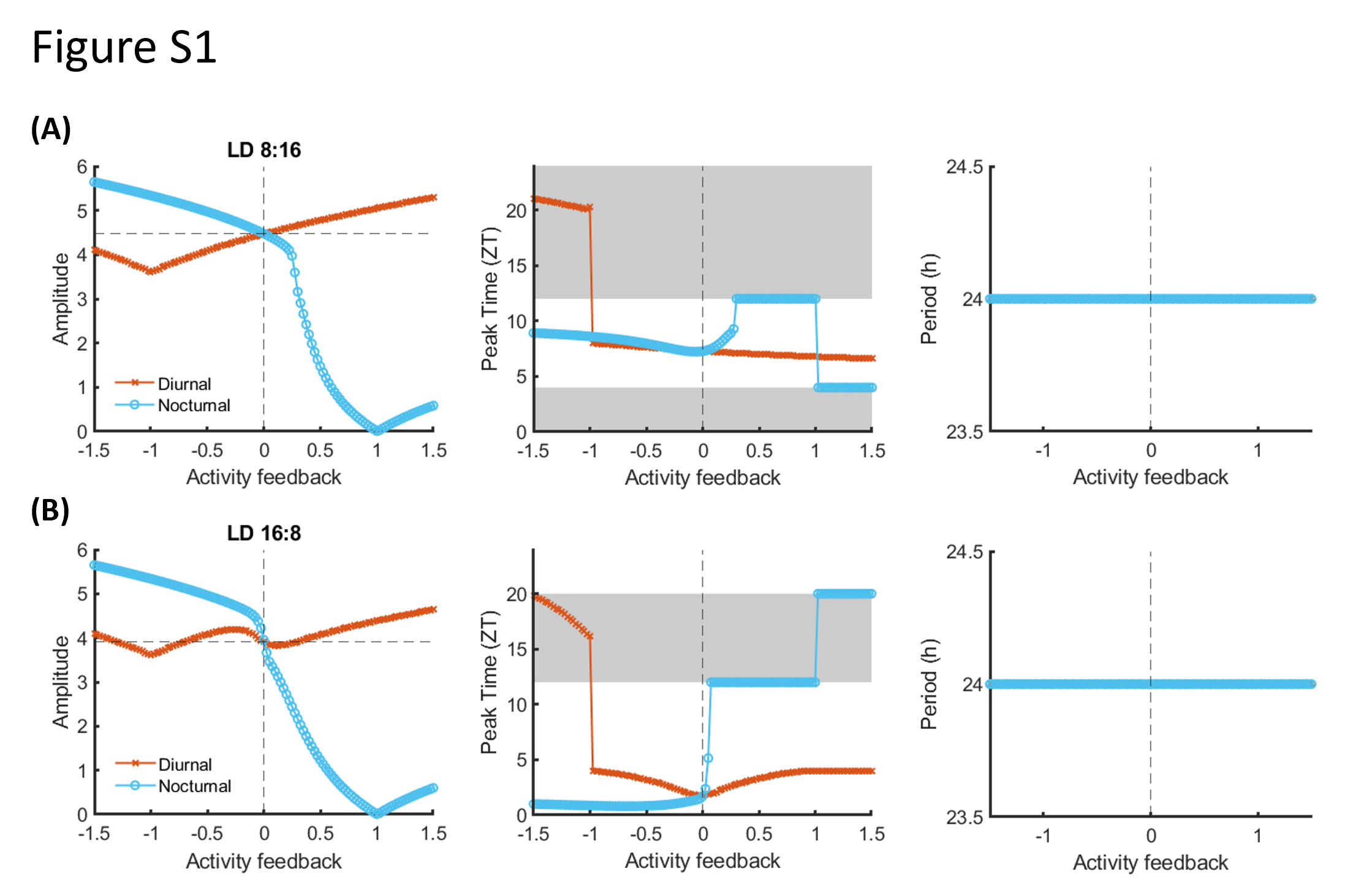

Supplement: sj-tif-1-jbr-10.1177_07487304241303554 – Supplemental material for Incorporating Physical Activity in a New Two-Oscillator Model of Circadian Activity in Nocturnal and Diurnal Mammals [file sj-tif-1-jbr-10.1177_07487304241303554.tif]

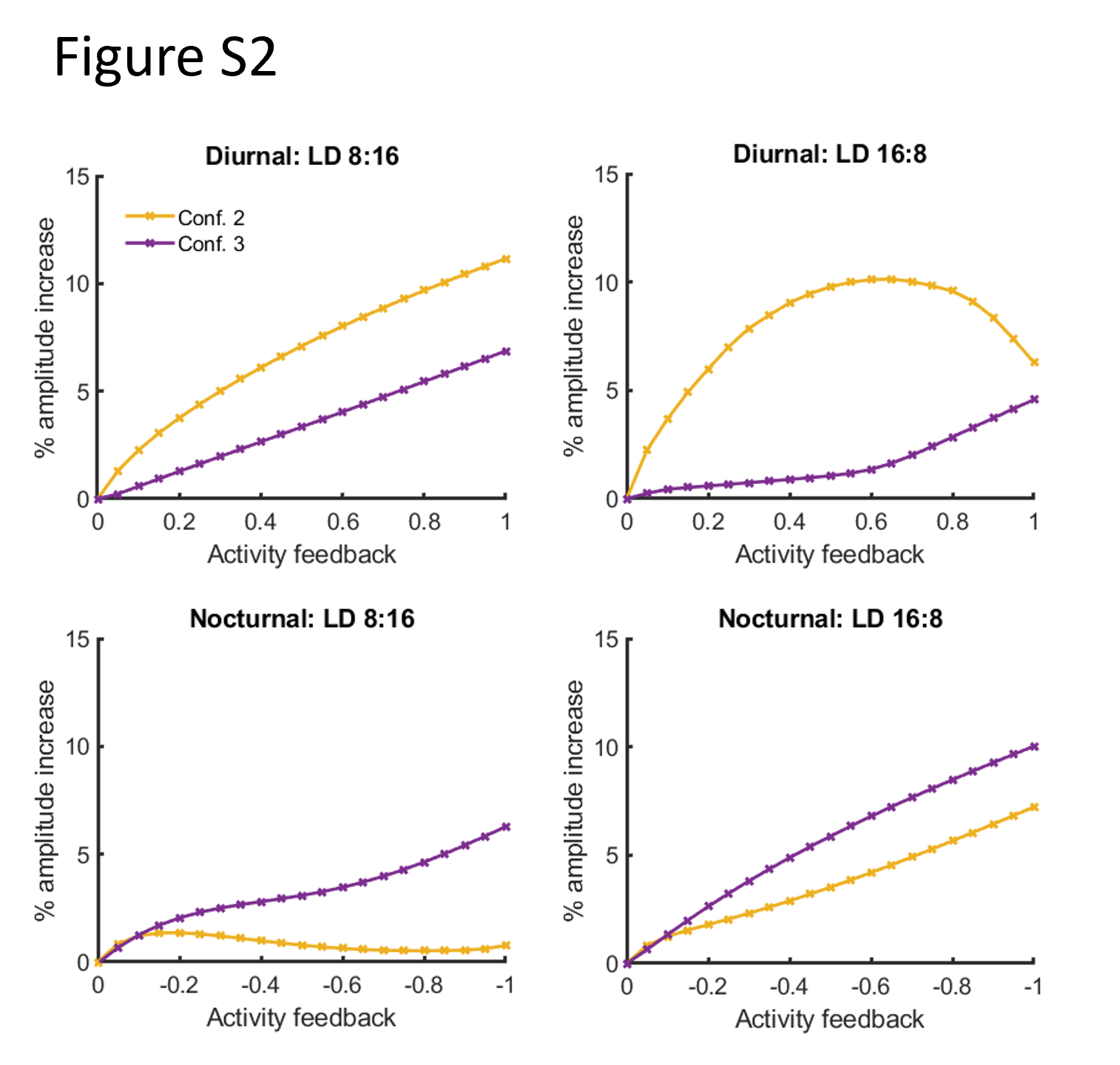

Supplement: sj-tif-2-jbr-10.1177_07487304241303554 – Supplemental material for Incorporating Physical Activity in a New Two-Oscillator Model of Circadian Activity in Nocturnal and Diurnal Mammals [file sj-tif-2-jbr-10.1177_07487304241303554.tif]
